# Patho-immune Mechanisms of Hypertension in HIV: a Systematic and Thematic Review

**DOI:** 10.1007/s11906-019-0956-5

**Published:** 2019-06-04

**Authors:** Sepiso K. Masenga, Benson M. Hamooya, Selestine Nzala, Geoffrey Kwenda, Douglas C. Heimburger, Wilbroad Mutale, Sody M. Munsaka, John R. Koethe, Annet Kirabo

**Affiliations:** 1 School of Medicine and Health Sciences, Mulungushi University, Livingstone, Zambia; 2 School of Health Sciences, Department of Biomedical Sciences, University of Zambia, Lusaka, Zambia; 3 Vanderbilt Institute for Global Health, Nashville, TN USA; 4 School of Public Health, Department of Epidemiology and Biostatistics, University of Zambia, Lusaka, Zambia; 5 Department of Medical Education Development, University of Zambia, Lusaka, Zambia; 6 School of Public Health, Department of Health policy and Management, University of Zambia, Lusaka, Zambia; 7 Division of Infectious Diseases, Vanderbilt University Medical Center, Nashville, TN USA; 8 Department of Medicine, Division of Clinical Pharmacology, Vanderbilt University Medical Center, 2215 Garland Avenue, P415C Medical Research Building IV, Nashville, TN 37232 USA; 9 Department of Molecular Physiology and Biophysics, Vanderbilt University, Nashville, TN USA

**Keywords:** HIV, Hypertension, Inflammation, Patho-immune mechanisms

## Abstract

**Purpose of Review:**

To systematically review recent findings on the role of immune cell activation in the pathogenesis of hypertension in people living with HIV (PLWH) and compare studies from Sub-Saharan Africa with what is reported in the USA and European literature according to guidelines of the Preferred Reporting Items for Systematic Reviews and Meta-Analyses.

**Recent Findings:**

PLWH have an increased risk for development of hypertension and cardiovascular disease. Chronic immune activation contributes to hypertension but the inflammatory milieu that predisposes PLWH to hypertension is poorly understood. We identified 45 relevant studies from 13 unique African countries. The prevalence of hypertension in PLWH on antiretroviral therapy (ART) and the ART-naive PLWH ranged from 6 to 50% and 2 to 41%, respectively. Interleukin (IL)-17A, interferon (IFN)-γ, and higher CD4^+^ T cell counts were associated with hypertension in ART-treated participants.

**Summary:**

Targeting adaptive immune activation could provide improved care for hypertensive PLWH. Further research is needed to characterize the inflammatory milieu contributing to hypertension in PLWH especially in African populations where the global burden of HIV is the highest.

## Introduction

The introduction of antiretroviral therapy (ART) has improved survival among people living with HIV (PLWH), but this success is accompanied by a high burden of cardiovascular disease [1–3]. Hypertension is a major risk factor to cardiovascular disease and its prevalence is higher in PLWH despite viral suppression by ART [4]. The mechanisms contributing to hypertension and cardiovascular disease in PLWH are not fully understood.

A heightened systemic inflammatory process including activation of the innate and adaptive immune systems contributes to the development of hypertension in the general population and in experimental animal studies [5–7]. However, it is unknown which innate and adaptive immune factors are most closely linked to hypertension in PLWH. At present, much of our understanding of the role of immune cell activation in the pathogenesis of hypertension comes from experimental animal and human studies in the HIV-negative context, but similar inflammatory biomarkers implicated in these HIV-negative studies have also been associated with hypertension in HIV infection. In this review, we present the current understanding on HIV, hypertension, and immune activation and how these may be related and highlight gaps and the need for further studies. We review studies conducted in Sub-Saharan Africa where the global HIV infection burden is the highest. We also performed a comparative analysis contrasting studies from Sub-Saharan Africa with what is reported in the USA and European literature.

The aim of our current study was to estimate the prevalence of hypertension in PLWH from African studies, identify inflammatory markers or markers of immune activation associated with hypertension in antiretroviral (ART)-treated HIV-positive individuals, and to explore the possible mechanistic interaction between immune activation, hypertension, and HIV in cohorts from Sub-Saharan Africa and contrast with European and US cohorts.

## Methods

We followed guidelines of the Preferred Reporting Items for Systematic Reviews and Meta-Analyses (PRISMA) [8, 9].

### Eligibility Criteria for Studies Included in the Review

We considered studies on HIV-positive human subjects with or without the HIV-negative population as controls. For exposure and outcome variables, we considered HIV infection as the exposure and our outcome measures were hypertension or blood pressure, inflammation, and/or immune activation. Only published articles in peer-reviewed journals were considered. Articles in languages other than English were not considered. We only included studies from Sub-Saharan African, USA, and European HIV-positive populations that reported on hypertension and HIV excluding studies that did not report HIV at all. We also excluded studies reporting opportunistic infections and other coinfections to reduce bias when deducting on the contribution of HIV on hypertension. In case of duplicate publications, the article with more complete data was included. Extracted information focused on study design, population, sample size, country where study was conducted, and details of findings with conclusions. The source of research articles was derived from Pubmed, Medline, google scholar, and google search. Dates of coverage were not specified to filter studies addressing hypertension and inflammation or immune activation in HIV owing to the scarcity of published studies. Other articles were identified from reference lists of related studies from the included study. Search terms that were used in databases employed the use of Boolean operators “AND,” “OR,” and “NOT” to refine our search by combining or limiting terms. The key terms used to search for articles were “hypertension,” “blood pressure,” “immune activation,” “inflammation,” “Africa,” and “HIV.”

Studies were screened based on abstract information that reported HIV or/and inflammation or immune activation and hypertension or blood pressure. A full article was then obtained and screened to ensure eligibility. The PRISMA 2009 Flow Diagrams for article identification, screening, eligibility, and inclusion in Sub-Saharan African and the Western populations are shown in Supplementary Figures 1 and 2 found at the following link: https://figshare.com/s/38ff86137d00153423ac. For data extraction, we used a data collection form that identifies the author, study, population, sample size, methods, findings, and limitations.

Risk of bias was assessed by two reviewers. For the criteria used to assess internal validity of included studies, we made use of the Cochrane Risk of Bias tool, paying attention especially to parts such as completeness of outcome data, selective outcome reporting, reporting bias, and any other bias that may affect the exposure-outcome relationship. For any disagreements between the reviewers over the risk of bias in particular studies, this was resolved by discussion, with involvement of a third review author where necessary.

### Data Synthesis

We did not conduct a meta-analysis. We only conducted a systematic thematic review due to the qualitative nature of our study and due to excessive heterogeneity of population, outcome, or methodology. To assist with the result and discussion write-ups, we constructed summary tables and provided a narrative synthesis (thematic or content analysis). This includes investigation of the similarities and the differences between the findings of different studies, as well as exploration of patterns in the data. Reasons for similarities and differences in the findings were also explored systematically. Studies were grouped based on reported parameters. We also considered how the results of studies might be affected by factors such as methodological differences, variable characteristics of the populations studied, or interventions investigated. Conceptual models were used to explore relationships and patterns from study findings and we related the findings to existing concepts from animal models to generate hypotheses and the need for further studies.

## Results

### Characteristics of the Studies

We performed a systematic review of studies on HIV, hypertension, and immunity from countries in Sub-Saharan Africa, USA, and Europe. For Sub-Saharan Africa studies shown in Table 1, we included 45 studies from 13 countries. Of the 45 studies, 27 were cross-sectional, 15 were prospective, and three were retrospective. Most of the studies (35) reported on both hypertension and HIV. The prevalence of hypertension was highest in the HIV-positive group on ART (ranging from 6.4 to 50.2%) followed by HIV-negative controls (13.7 to 44%). ART-naive patients recorded the lowest prevalence ranging from 2 to 41% by group comparisons. The prevalence differences of groups within studies were highest between ART treated and ART-naive HIV-positive groups (35.7) followed by ART treated and HIV-negative group (18.7) with the least difference in magnitude between HIV negative and ART-naive groups.

**Table 1:** Characteristics of studies included in the Sub-Saharan African studies

Study characteristics	Number (*n*) or percentage (%)
Number of studies included	45
Number of African countries where the studies were conducted	13
Number of types of studies	3
Studies reporting on inflammation *plus* hypertension *plus* HIV only	5
Studies reporting on hypertension *plus* HIV only	35
Studies reporting on HIV *plus* immune activation/inflammation	5
Studies reporting on inflammation *plus* hypertension in African populations	0
Overall prevalence of HTN in PLWH on ART (% range)	6.4–50.2
Overall prevalence of HTN in ART-naive PLWH (% range)	2–41
Overall prevalence of HTN in HIV-negative population (% range)	13.7–44
HTN prevalence percentage differences within studies, *range* (│*magnitude*│)	
PLWH on ART *minus* ART naive	− 12.3 to 23.4 (│35.7│)
PLWH on ART *minus* HIV negative	− 6.3 to 12.4 (│18.7│)
HIV negative *minus* ART naive	− 5.3 to 11 (│16.3│)

HTN, hypertension; HIV, human immune deficiency virus; ART, antiretroviral therapy; PLWH, people living with HIV

When we included USA and European studies, shown in Supplementary Table 1 found at the following link: https://figshare.com/s/38ff86137d00153423ac, the global prevalence of hypertension in PLWH ranged from 4 to 57% (van Ziest et al. 2017) [10]. Hypertension was more prevalent in HIV uninfected (71%) versus HIV infected (57%) in a large longitudinal study by Armah et al. [11]. HIV-infected veterans with HIV-1 RNA ≥ 500 copies/mL or CD4 count < 200 cells/μL had a significantly higher prevalence of elevated IL-6 and D-dimer after adjusting for comorbidities and had significantly higher prevalence of elevated sCD14 compared to uninfected veterans. Manner et al. [12] reported prevalence of 35% hypertension in HIV-infected individuals and the prevalence did not change during the follow-up time (3.4 ± 0.8 years). CD4 T cell count < 50 cells/μL and increased duration of ART were independent predictors of sustained hypertension throughout the study period. Older age, male gender, BMI > 25 kg/m^2^, and baseline CD4 cell count ≥ 200 cells/μL were also independent predictors of sustained hypertension. Markers of microbial translocation predicted hypertension in HIV-infected individuals. Manner et al. found that both LPS and sCD14 independently predicted subsequent blood pressure levels after adjustment for age and gender [12]. These results suggest that ART may act as a contributing factor to inflammation and the increased prevalence of hypertension in the PLWH.

### Higher Levels of IL-17A, IFN-γ, and CD4^+^ T Cells Are Associated with Hypertension in HIV

We found that among studies reporting on both inflammation, immunity, and hypertension in HIV (Table 2), higher levels of IL-17A, IFN-γ, [13••], and CD4^+^ T cell count (Peck et al.) [4] were significantly associated with hypertension in ART treated HIV-positive individuals. The cross-section study by Chepchirchir et al. reported that females were more likely to have higher IL-17A levels than males and IL-17A was affected by BMI but not stress levels, ART, World Health Organization (WHO) stage, and CD4^+^ count [13••]. However, inflammatory cytokines IL-2, IL-6, IL-8, tumor necrosis factor alpha (TNF-α), and anti-inflammatory cytokines IL-4 and IL-10 were not associated with hypertension. The cross-section study by Peck et al. reported higher cases of hypertension among PLWH on ART [28.7% (43/150)] compared to ART-naive participants [5.3% (8/151)]; however, the prevalence was higher in HIV-negative patients [16.3% (25/153)] compared to ART-naive HIV-positive patients. The inflammatory markers, C-reactive protein (CRP), and IL-6 in the prospective study by Fourie et al. where the three participant groups had comparable blood pressures did not differ between HIV positive on ART, ART naive, and HIV-negative groups. In another prospective study by Okello et al., blood pressure increases were reported in the first 6 months of ART initiation then plateaued. Traditional risk factors including older age, male gender, African-American, higher body mass index (BMI), central obesity, previous cardiovascular events, chronic kidney disease, family history of hypertension and cardiovascular disease, diabetes, and dyslipidemia rather than immune activation were associated with incident hypertension in this study. Borkum et al. reported high levels of inflammation and non-dipping blood pressure [14••].

**Table 2:** Characteristics and findings of studies reporting on inflammation, hypertension, and HIV

Author/	Type of study, country, and population	Sample size and subjects	Key findings	Limitations/notes/conclusion
Chepchirchir et al. 2018 [13••]	Cross-section study conducted in Kenya among PLHIV	126 HIV-positive with and without hypertension	• HTN prevalence was 23.2% • IL-17A was associated with hypertension in HIV {HTN 75.51 ± 84.89 versus normotensives 42.23 ± 64.40; CI 6.81–59.77, *p* < 0.001 and females were more likely to have higher IL-17A levels than males in HIV (*p* < 0.001) • IL-17A was affected by BMI *r*(124) = 0.223 *p* = 0.070 but not stress levels, ART, WHO stage and CD4+ count • IFN-γ {HTN 6.87 ± 35.66; normotensives 0.00 ± 0.00 CI − 1.96–15.71, *p* = 0.003} correlated negatively with hypertension status (*r*_s_ = − 0.217, *p* < 0.015) • IL-4,-2,-6,-8,-10, TNFα were not associated with hypertension	• Monitoring and analysis of cytokine expression may help to predict patients’ pathways in their response to cART therapy and risk of metabolic disorders • IFNγ is potentially useful in determining risk of developing hypertension in this population • Mechanism of interaction between hypertension and inflammation or immune activation not very explicit
Peck et al. 2014 [14••]	Cross-sectional study conducted in Tanzania among PLHIV	454 participants: HIV+ on ART, ART-naive HIV+ and HIV-negative controls	• Prevalence of HTN in HIV negative was 16.3% (25/153) • Prevalence of HTN in HIV+ on ART was 28.7 (43/150) was the highest (*p* = 0.01) among the groups with higher odds HTN even after adjustment (odds ratio (OR) = 2.19 (1.18 to 4.05), *p* = 0.01) • Prevalence of HTN in ART-naive HIV+ was 5.3% (8/151), and lowest among group (*p* = 0.003) with lower odds of HTN even after adjustment (OR = 0.35 (0.15 to 0.84), *p* = 0.02) • The prevalence HTN was lowest in group with the lowest average CD4+ T cell count (HIV-infected ART naive) and highest in the group in which the CD4+ T cell count had been low and had then been reconstituted in the setting of ART • In the HIV+ group, higher CD4+ T cell counts were associated with more hypertension and higher blood pressures • Age, vigorous work, current alcohol use, and BMI were all associated with hypertension • Only PI use was associated with HTN • Among the HIV-infected adults with HTN, 75% were undiagnosed, 85% were untreated, and > 95% were uncontrolled	• HIV-infected adults with hypertension were rarely aware of their diagnosis but often have evidence of kidney disease • It was unknown whether chronic inflammation accelerates HTN • High prevalence of hypertension among HIV-infected adults on ART could be related to dysregulated inflammation due to immune reconstitution • Mechanism of interaction between hypertension and inflammation or immune activation not explicit
Fourie et al. 2015 [15]	Prospective study conducted in South Africa	309 participants: 66 HIV+ on ART, 78 (ART-naive HIV+ and165 HIV-negative controls	• The inflammatory markers (CRP and IL-6) did not differ between the three groups • Endothelial activation was not accompanied by increased inflammation • BP comparable among groups	• Mechanism of interaction between hypertension and inflammation or immune activation not explicit • Several cytokines not reported
Okello et al. 2016 [16]	Prospective cohort study conducted in Uganda	536 HIV positive initiating ART	• Systolic BP increased by 9.6 mmHg/year (95% CI 7.3–11.8) in the first 6 months of ART, then plateaued • Male gender, overweight, and a CD4 count < 100 cells were associated with incident hypertension	• Blood pressure increases early after ART initiation in Ugandans. Traditional risk factors, rather than immune activation were associated with incident hypertension in this population • Only sCD14, sCD163 (immune activation markers), and IL-6 (inflammatory marker) were examined • Mechanism of interaction between hypertension and inflammation or immune activation not explicit
Borkum et al. 2017 [17]	Cross-sectional study conducted among South African blacks	67 HIV-positive participants on ART > 5 years	• Prevalence of non-dipping BP was 65% • High levels of inflammation (hsCRP) • There was no association on multivariable analysis between dipping status and traditional risk factors for non-dipping BP, such as obesity, autonomic dysfunction, and older age	• 91% (*n* = 61) were females • Inflammation was only assessed using hsCRP • Mechanism of interaction between hypertension and inflammation or immune activation not explicit

PLHIV, people living with HIV; HTN, hypertension; HIV, human immunodeficiency virus; BP, blood pressure; CRP, C-reactive protein; ART, antiretroviral therapy; PI, protease inhibitors; IL, interleukin; BMI, body mass index; CI, confidence interval; TNFα, tumor necrosis factor alpha; IFNγ, interferon gamma; WHO, World Health Organization; cART, combinational ART; sCD14, soluble CD14; sCD163, soluble CD163; hsCRP, high-sensitivity C-reactive protein

### Prevalence of Hypertension Is Higher in ART-Treated PLWH

As shown in Table 3, hypertension prevalence was mostly higher in men than women [15–17] and higher among ART treated versus ART naive (28.7% vs 5.3%; 17% vs 2%; 30 vs 21.9% and 38% vs 19%, respectively) [17, 18]. However, one study [22] reported contradictory findings (ART vs ART naive, 12.3 vs 19%). In another study, [17] reported higher prevalence of hypertension in ART treated group (28.7%) compared to the HIV-negative controls (16.3%) but two studies [22, 25] reported the opposite (41% vs 44% and 12.3% vs 13.7%, respectively). Peck et al. reported higher prevalence of hypertension in HIV negative vs ART-naive HIV-positive group (16.3% vs 5.3%) [17]. On the contrary, Ogunmola et al. reported lower prevalence of hypertension in HIV negative vs ART-naive HIV-positive group (13.7% vs 19%, respectively). Association between hypertension and traditional risk factors including specific ART regimens varied between studies.

**Table 3:** Characteristics and findings of studies reporting on hypertension and HIV

Author/	Type of study, country and population	Sample size and subjects	Key findings	Limitations/notes/conclusion
Bloomfield et al. 2011 [18]	Retrospective study conducted in Kenya among PLWH	12,194 HIV-positive participants	• HTN prevalence among men 11.2% and women 7.4%, overall 8.7% • Age, overweight/obesity, longer duration on PI were not associated with HTN • HTN more common in younger HIV vs older • Higher HTN cases associated with patients with higher CD4 in men than women but blunted HTN occurred with older age	Immune mechanisms not addressed explicitly
Julius et al. 2011 [19]	Cross-sectional study conducted in South Africa among PLWH on ART	304	Prevalence of HTN was 19.1%. 23.9% in men and 17.7% in women (95% confidence interval (CI) 14.7–23.5), *p* = 0.10)	Immune mechanism not addressed
Diouf et al. 2012 [20]	Cross-section study conducted in Senegal among PLWH	242 HIV	• 28.1% had hypertension • ART duration not associated with HTN • Longer exposure to LPV/r was associated with a reduced risk of hypertension	Higher hypertension observed for male patients with CD4 count above 200 cells/μL, but differences were not statistically significant
Ngatchou et al. 2013 [21]	Cross-sectional study conducted in Cameroon among PLWH	204 participants consisting of 108 HIV ART naive vs 96 HIV negative	Prevalence of HTN in HIV was 41% and HIV negative 44%	Immune mechanism or inflammation as it relates to hypertension was not addressed
Parikh et al. 2013 [22]	Cross-sectional study conducted in Uganda and Zimbabwe among PLWH	3316	Prevalence of systolic and diastolic hypertension was 21.3% and 19.0% for older adults; and 9.2% and 3.5% for younger adults with HIV (both, *p* < 0.001)	Immune mechanism or inflammation as it relates to hypertension was not addressed
Ekali et al. 2013 [23]	Cross-sectional study conducted in Cameroon among PLWH	143	SBP and DBP increased with duration on ART. HTN was associated with longer duration on ART	Immune mechanism or inflammation as it relates to hypertension was not addressed
Muhammad et al. 2013 [24]	Cross-sectional study conducted in Nigeria among PLWH	200 HIV+ ART and ART-naive participants	HTN prevalence was 17% in ART and 2% in ART naive (*p* < 0.001) HAART was associated with HTN	Immune mechanism or inflammation as it relates to hypertension was not addressed
Mateen et al. 2013 [25]	Cross-sectional study conducted in Uganda among PLWH initiating ART	5563	HTN prevalence was 27.9%	Immune mechanism or inflammation as it relates to hypertension was not addressed
Botha et al. 2014 [26]	Prospective study conducted in South Africa among PLWH	137 participants: 66 HIV+ on ART and 71 HIV+ ART-naive participants	HIV+ on ART had higher pulse pressure (13.3%; *p* = 0.004), systolic blood pressure (4.5%; *p* = 0.029), and CD4 cell count (9.2%; *p* = 0.009) levels over 5 years	Immune mechanism or inflammation as it relates to hypertension was not addressed
Ogunmola et al. 2014 [27]	Cross-section conducted in Nigeria among PLWH	403 participants. Groups: HIV-negative controls, HIV+ on ART, and ART-naive HIV+	• Prevalence was 13.7% in HIV negative, 19.0% in HIV+ ART naive, and 12.3% in HIV-positive ART subjects • Multivariate regression analysis showed no relationship between hypertension and HIV status (*p* = 0.293) or ART status (*p* = 0.587) but only with BMI	Immune mechanism or inflammation as it relates to hypertension was not addressed
Shaffer et al. 2014 [28]	Prospective study; randomized, open-label ART trials among 7 African countries (South Africa; Kenya; Zimbabwe, Botswana, Zambia, Malawi, and Uganda)/population included HIV+ women only with immunocompromised CD < 200	741	Over 144 weeks NVP compared to LPV/r group had greater mean increases in BP (diastolic BP 22.7% vs. 6.5%)	Immune mechanism or inflammation as it relates to hypertension was not addressed
Sawadogo 2014 [29]	Cross-sectional study conducted in Burkina Faso among PLWH on ART	400 participants	Hypertension prevalence was 12.0%	Immune mechanism or inflammation as it relates to hypertension was not addressed
Kagaruki et al. 2014 [30]	Cross-section study conducted in Tanzania	671 participants HIV+ on ART and ART naive	• The prevalence of hypertension was 26.2% and was high among those on ART (30.0% vs 21.9%, *p* = 0.010) • Aged > 40 years (AOR = 2.52, 95% CI 1.37–4.63), abnormal waist circumference (AOR = 2.37 95% CI 1.13–5.00), overweight/obesity (AOR = 2.71, 95% CI 1.26–5.84), and male sex (AOR = 1.17, 1.02–4.20) were the predictors of hypertension among patients on ART while raised total cholesterol (AOR = 1.47 (1.01–2.21) and being aged > 40 years (AOR = 3.42, 95% CI 2.06–5.70) were predictors for hypertension among ART-naive patients	Immune mechanism or inflammation as it relates to hypertension was not addressed
Abrahams et al. 2015 [31]	Prospective study from South Africa in HIV+ women	103 participants	• Systolic and diastolic blood pressure increased significantly and the proportion of participants with hypertension increased from 3.9 to 15.5% (*p* < 0.001) • Long-term exposure to ART, increased in hypertension • Stavudine and efavirenz and nevirapine were significantly associated with hypertension	Immune mechanism or inflammation as it relates to hypertension was not addressed
Okello et al. 2015 [32]	Prospective study in Uganda	3389 participants; HIV+ initiating ART	• 13% incidence of HTN • Male gender (AHR 1.88, 95% CI 1.49–2.39), increasing age (AHR 1.36, 95% CI 1.02–1.82 for those > 40 years compared to those aged 30 years or less), nadir CD4 count (AHR 0.77, 95% CI 0.60–0.99 and AHR 0.64 95%CI 0.41–1.00 for a nadir CD4 cell count 100–350 and > 350 cells/mm^3^ compared to < 100 cell/mm^3^, respectively), and high baseline body mass index (AHR 2.50, 95% CI 1.56–4.01 for those with a BMI ≥ 30 kg/m^2^ versus normal BMI) were independently associated with increased risk of hypertension • Neither use of TDF versus AZT, nor use of NVP versus EFV was associated with risk of hypertension in the multivariate model • Inverse relationships between hypertension risk and CD4 count nadir. Data suggest that immunosuppression and/or viral burden play a role in promoting early vascular damage as evidenced by the association of a low CD4 cell count with subclinical atherosclerotic damage, a processor of hypertension	• Results are the first to document high rates incident hypertension during longitudinal follow-up among initially normotensive PLHIV initiating ART • Particularly notable was the magnitude of hypertension incidence in younger groups • Potential mechanisms for increased incidence of hypertension postulated in this paper include HIV-associated chronic inflammation, immune suppression, endothelial activation, and dysfunction, as well as the direct infection of arterial vascular smooth muscle cells by HIV • Alternatively, incident hypertension in this setting may reflect a “return to health” as individuals gain weight during ART, particularly with advanced pre-ART disease stages Limitation: smoking status and laboratory results of blood glucose, serum creatinine, serum electrolytes, and serum lipids were not available for most patients; therefore, the contribution of clinical factors known to be associated with hypertension such as diabetes mellitus, chronic kidney disease, and hyperlipidemia could not be assessed
Dimala et al. 2016 [33]	Cross-sectional study conducted in Cameroon among PLWH	200 HIV+ ART and ART-naive participants	• Prevalence of HTN in patients on HAART was twice (38%; 95% CI 28.5–48.3) that of the HAART-naive patients (19%; 95% CI, 11.8–28.1), *p* = 0.003 • CD4 cell count (mean ± SD, cells/μL) of ART 501 ± 225 was higher than the ART naive 197 ± 160 ˂ 0.001 • HTN was associated with older age and male gender, in the HAART group	Immune mechanism or inflammation as it relates to hypertension was not addressed
Feigl et al. 2016 [34]	Prospective study from South Africa among PLWH	505 participants: HIV+ on ART, ART-naive HIV+ and HIV-negative controls	• DBP increased in ART naive but SBP reduced compared to the HIV-negative group • SBP in the HIV+ on ART− group showed a significant decline	They could not control for a range of potential time-variant confounders in their analysis due to lack of data
Kalyesubula et al. 2016 [35]	Retrospective study from Uganda among PLWH	1996 HIV+ on ART and ART-naive participants	• Prevalence of hypertension was 20.9% (418/1996) rising from 16.9% in 2009 to 32.3% in 2013 • Patients > 50 years of age had 3.12 times the odds of hypertension compared with patients aged 20–29 years (95% CI 2.00 to 4.85) • Men had 1.65 times the odds of hypertension compared with women (95% CI 1.34 to 2.03) and patients with a BMI of 35–39 kg/m^2^ had 3.93 times the odds of hypertension compared with patients with a BMI < 25 kg/m^2^ • Patients with a WHO disease staging of 3 or 4 had 0.60 times the odds of hypertension compared with patients with stage 1 or 2 (95% CI 0.46 to 0.76)	Analysis did not distinguish ART and ART-naive patients as this can confound results
Kwarisiima et al. 2016 [36]	Cross-sectional study from Uganda	94,274 participants HIV+ and HIV negative	• Hypertension prevalence was 11% among HIV-positive individuals • 79% of patients were previously undiagnosed, 85% were not taking medication, and 50% of patients on medication had uncontrolled blood pressure • Multivariate predictors of hypertension included older age, male gender, higher BMI, lack of education, alcohol use, and residence in Eastern Uganda • HIV-negative status was independently associated with higher odds of hypertension (OR 1.2, 95% CI 1.1–1.4). Viral suppression of HIV did not significantly predict hypertension among HIV positives • The prevalence of HTN was greater among HIV-negative adults (14%) than among HIV-positive adults (11%) • Among HIV-positive adults with HTN, 20% reported prior knowledge of HTN, and 14% reported taking medication • Similar to the overall population, 46% of HIV-positive adults with HTN achieved BP control with antihypertensive medications	Immune mechanism or inflammation as it relates to hypertension was not explicitly addressed
Njelekela et al. 2016 [37]	Cross-sectional study from Tanzania among PLWH	34,111 HIV-positive ART naive only	• Prevalence of hypertension was found to be 12.5% • Risk of hypertension was 10% more in male than female patients • Patients aged ≥ 50 years had more than 2-fold increased risk for hypertension compared to 30–39-year-old patients • Overweight and obesity were associated with 51% and 94% increased risk for hypertension compared to normal weight patients • Low CD4^+^ T cell count, advanced WHO clinical disease stage, and history of TB were associated with 10%, 42%, and 14% decreased risk for hypertension	Immune suppression and history of TB were associated with lower risk for hypertension
Osegbe et al. 2016 [38]	Cross-section study from Nigeria	283 HIV+ on ART, ART naive and HIV-negative participants	• SBP in HIV+ ART was higher (124.9 ± 20.7 mmHg) compared to HIV+ ART naive which was higher than (121.5 ± 20.7) HIV negative (114.8 ± 11.7 *p* = 0.001) • hsCRP = 2.9 (1.4–11.6), *p* = 0.002 were higher among the HIV-naive subjects • Higher prevalences of the risk factors were noted among the HIV-treated subjects except low HDL-C (*p* < 0.001) and hsCRP (*p* = 0.03) which were higher in the HIV-naive group	Immune mechanism or inflammation as it relates to hypertension was not addressed; however, hsCRP was higher among ART-naive patients
Divala et al. 2016 [39]	Cross-section study from Malawi	952 HIV+ (95% were on ART and 5% ART naive)	• Prevalence was 23.7% (95%-confidence interval 21.1–26.6; rural 21.0% vs. urban 26.5%; *p* = 0.047) • Hypertension diagnosis was associated with increasing age, higher body mass index, presence of proteinuria, being on regimen zidovudine/lamivudine/nevirapine and inversely with World Health Organization clinical stage at ART initiation	Immune mechanism or inflammation as it relates to hypertension was not addressed
Magodoro et al. 2016 [40]	Cross-section study conducted in Zimbabwe among PLWH	1033 participants	• Hypertension prevalence was 10.2%	Studies had other substantial burden of comorbid non-communicable diseases among HIV-infected patients in a high HIV and low-income setting
Nduka et al. 2016 [41]	Prospective study from Nigeria	303 participants—229 on ART and 74 ART naive	In this propensity score-matched sample, the estimated average treatment effects on the treated (ATT) for the effects of antiretroviral therapy on systolic (7.85 mmHg, 95% CI 3.72 to 15.68) and diastolic blood pressure (7.45 mmHg, 95% CI 4.99 to 13.61) were statistically significant (*p* < 0.001 for each)	There is a high probability that the epidemiological association between antiretroviral therapy and increased blood pressure be causal in nature among people living with HIV in Sub-Saharan African settings
Bauer et al. 2017 [42]	Prospective study conducted in Lusaka, Zambia among PLWH	896 cohort HIV+ on ART participants	• 98 (10.9%) had HBP, and 57 (6.4%) had HTN • Increasing age (10-year increase in age: adjusted odds ratio [AOR] = 1.50; 95% confidence interval [CI] 1.20–1.93), male sex (AOR = 2.33, 95% CI 1.43–3.80), and overweight/obesity (AOR = 4.07; 95% CI 1.94–8.53) were associated with HBP • Among 66 patients with HBP, 35 (53.0%) reported awareness of the condition • Pre-ART CD4^+^ count was not associated with HBP HBP tends to become more common after ART initiation and may cause HTN	Coinfection with HBV, HBP was defined as a single elevated measurement, and BP was measured only once at each time point. This may have inflated the number of patients truly needing treatment, as only 58% of patients with HBP were confirmed to have HTN These data demonstrate that although integration of BP screening and management in HIV care settings was feasible in Zambia, virtually no patient had optimal management of HBP
Kazooba et al. 2017 [43]	Cross-section study from Uganda	1024 HIV+ ART	• Hypertension prevalence was 22.6% • Protease inhibitor (PI) containing regimens were significantly associated with, hypertension • Men had significantly higher mean SBP (*p* = 0.004) • Increasing age was significantly associated with higher means of SBP, DBP • Increasing intensity of physical activity was significantly associated with lower; SBP, DBP	Immune mechanism or inflammation as it relates to hypertension was not addressed
Mayanja et al. 2017 [44]	Prospective study from Uganda	1095 HIV+ on ART individuals	• Patients on non-PI regimens had higher mean diastolic hypertension than patients on PI regimens, *p* < 0.001 • Prevalence of hypertension was 14.5%	Immune mechanism or inflammation as it relates to hypertension was not addressed
Okpa et al. 2017 [45]	Cross-section study from Nigeria	112 HIV+ and 309 HIV-negative participants	• The prevalence of HTN was 19.5% among HIV-infected persons • The prevalence of pre-HTN in HIV-positive and HIV-negative participants was 34.5% and 38.9%, respectively • The higher prevalence of HTN in HIV patients has been shown to be associated with the use of ART • In our study, the risk factors that were associated with HTN in HIV-infected patients and the HIV-N controls were older age, increased weight and BMI, and presence of obesity. Male sex was associated with HTN in the HIV-infected population, but this was, however, not so in the general population • Male sex and duration of exposure to ART and CD4 count levels > 200 cells/mm^3^ were associated with HTN in HIV-infected patients, whereas the absence of family history of HTN was significantly associated with HTN in both groups • The mean age, weight, and BMI were significantly higher in HIV patients with HTN as compared to those without HTN, *p* < 0.05 • A proportion of HIV patients with a family history of HTN and obesity had HTN, and these were statistically significant (*p* < 0.05) • Also, the mean waist-hip ratio, duration of illness, exposure to ART, and CD4 count levels were higher in HIV patients with HTN as compared to those without HTN, but these were not statistically significant	Diet and sodium intake of the participants and renal function of the participants which have been shown in several studies to have effect on BP were not looked at in our study Immune mechanism or inflammation as it relates to hypertension was not addressed
RodrõÂguez-Arbolõ et al. 2017 [46]	Prospective study from Tanzania	955 HIV+ initiating ART	• 111 (11.6%) were hypertensive at recruitment • 80 (9.6%) of them developed hypertension during a median follow-up of 144 days from time of enrolment into the cohort [incidence rate 120.0 cases/1000 person-years, 95% confidence interval (CI) 97.2 ± 150.0]	Traditional cardiovascular risk factors predicted incident hypertension, but no association was observed with immunological or ART status. These data support the implementation of routine hypertension screening and integrated management into HIV programs in rural Sub-Saharan Africa
Isa et al. 2017 [47]	Prospective study from Nigeria/	Follow-up from initiating HIV+ ART and naive	• Prevalence of hypertension at enrolment was 19.3% (95% CI 17.6–20.9%) • Age (*p* < 0.001), male sex (*p* = 0.004), and body mass index (BMI) (*p* < 0.001) were independent risk factors for hypertension • 12 months after initiating ART, a further 31% (95% CI 17.6–20.9%) had developed hypertension. Total prevalence at that point was 50.2% • Hypertension among those on ART was associated with age (*p* = 0.009) and BMI (*p* = 0.008), but not with sex • There were no independently significant associations between hypertension and CD4^+^ counts, viral load, or type of ART	Hypertension is common in HIV-infected individuals attending the HIV clinic. Patients initiating ART have a high risk of developing hypertension in the first year of ART
Magande et al. 2016 [48]	Case control study from Zimbabwe among HIV+ on ART	300 (152 controlled HTN, 152 uncontrolled HTN)	• Adding salt to dishes regularly AOR = 5.69 (3.19–10.16), body mass index (BMI) above 25 kg/m^2^ AOR = 2.81 (1.60–4.91) and history of elevated blood pressure in previous year AOR = 2.34 (1.33–4.13) were independent risk factors for uncontrolled hypertension • Independent protective factors were duration more than 2 years since HIV diagnosis AOR = 0.58 (0.35–0.95), duration less than 5 years since hypertension diagnosis aOR = 0.50 (0.30–0.83) and walking or cycling as a means of transport AOR = 0.27 (0.16–0.48)	Salt HTN interaction attributed to water retention resulting in high intravascular volumes. Immune mechanism or inflammation as it relates to hypertension was not addressed
Chepchirchir et al. 2018 [13••]	Cross-section study from Kenya among HIV+	297	The prevalence of hypertension was found to be 23.2%	Hypertension is a highly prevalent comorbidity in HIV patients. The risk factors include prolonged use of ART as well as increased body mass index. The effects of hypertension on HIV progression include low CD4^+^ T cell counts which complicate the underlying immunosuppression
Okello et al. 2017 [49]	Cross-section study from Uganda among PLWH	577 HIV-infected and 538 matched HIV-uninfected participants	• HIV infection was associated with 3.3 mmHg lower systolic BP (1.2–5.3 mmHg), 1.5 mmHg lower diastolic BP (0.2–2.9 mmHg), 0.3 m/s lower pulse wave velocity (0.1–0.4 mmHg), and 30% lower odds of hypertension (10%–50%) • Body mass index mediated 25% of the association between HIV and systolic BP	HIV infection was inversely associated with systolic BP, diastolic BP, and pulse wave velocity Immune mechanism or inflammation as it relates to hypertension was not addressed
Mukeba-Tshialala et al. 2017 [50]	Cross-section study from the Republic of Congo	592 HIV-uninfected and 445 HIV-infected patients	• 11.5% of HIV-infected patients had an average blood pressure suggesting hypertension versus 10.6% of HIV uninfected (*p* = 0.751) • But in absolute value, HIV-infected patients had a median of diastolic blood pressure of 90 mmHg versus 85 mmHg of HIV uninfected (*p* < 0.001)	Immune mechanism or inflammation as it relates to hypertension was not addressed
Mathabire Rücker et al. 2018 [51]	Cross-section from Malawi among PLWH	379 HIV-infected patients on ART and 356 controls participated	• Among HIV patients, the prevalence of hypertension was 19.5% (95% CI 15.6–23.6), of which 60.3% (*n* = 44) was previously undiagnosed • Among controls, 25.8% (95% CI 21.6–30.7) prevalence of HTN and 37.0% of controls with elevated blood pressure	Immune mechanism or inflammation as it relates to hypertension was not addressed

DBP, diastolic blood pressure; SBP, systolic blood pressure; PLWH, people living with HIV; HTN, hypertension; HIV, human immunodeficiency virus; BP, blood pressure; ART, antiretroviral therapy; PI, protease inhibitors; BMI, body mass index; CI, confidence interval; cART, combinational ART; TDF, Tenofovir; AZT, Zidovudine; NVP, Nevirapine; EFV, Efavirenz; HAART, highly active antiretroviral therapy; LPV/r, lopinavir/ritonavir; TC, total cholesterol; HDL-c, high-density lipoprotein cholesterol

### ART Treatment in PLWH Is Associated with Higher Inflammation

As shown in Table 4, we found that PLWH on ART had higher inflammatory markers including IL-6, CRP, intracellular adhesion molecule 1 (ICAM-1), and vascular adhesion molecule 1 (VCAM-1) compared with HIV-negative individuals (Fourie et al.) [55]. Intermediate monocytes (CD14^+^16^+^) increased with viremia in immune-compromised patients and microbial translocation was a major force driving chronic systemic inflammation in HIV-positive individuals on ART.

**Table 4:** Studies on HIV and immune activation/inflammation

Author/	Type of study/populations/country/study period	Sample size/methods	Key findings	Limitations/notes
Fourie et al. 2011 [52]	Sub-study nested in the larger longitudinal, multinational PURE study in South Africa among PLWH/HIV+ and HIV−	600 HIV+ and HIV-negative controls	• HIV+ showed higher IL-6, CRP, ICAM-1, and VCAM-1 levels compared to HIV negative • Accelerated vascular aging and probable early atherosclerosis in the older HIV-infected participants • HIV-1 Tat protein (therefore HIV itself) induced the expression of ICAM-1 and VCAM-1	Immune mechanism or inflammation as it relates to hypertension was not addressed
Cassol et al. 2010 [53]	Cross-section study conducted from South Africa	90 participants 10 HIV-negative controls, 20 HIV+ on ART, and 60 ART-naive HIV+	• CD14^+^16^+^ positively correlated with HIV-1 viremia • IL-6 increased in presence of OPIs • sCD14 and TNF correlated with plasma LPS levels and remained elevated in ART • Microbial translocation is a major force driving chronic inflammation in HIV-infected Africans receiving cART	All participants were immunocompromised with CD4 counts of < 200 cells/μL Immune mechanism or inflammation as it relates to hypertension was not addressed Prevention of monocyte activation may be especially effective at enhancing therapeutic outcomes
Fourie et al. 2011 [52]	Cross-section study from South Africa	300 ART-naive HIV+ vs 300 HIV negative	• Suggestion of inflammatory injury of the endothelium	Contribution of innate and adaptive immune system were not included or addressed
			• Indication of accelerated vascular aging and probable early atherosclerosis in the older HIV-infected participants	
Canipe et al. 2014 [54]	Zambia/pilot study 12 months prospective	33 HIV+ on ART for 12 months	• Biomarkers of increased microbial translocation (lipopolysaccharide binding protein, endotoxin core IgG and IgM, and soluble CD14) were associated with high levels of systemic inflammation before and after initiation of ART, suggesting that impaired gut immune defenses contribute to innate immune activation in this population	All the HIV-infected adults in the study were undernourished
Siedner et al. 2016 [55]	Prospective study from Uganda	105 HIV positive initiating ART	• Persistent immune activation (sCD14 level and kynurenine-tryptophan ratio) and inflammation (IL-6) despite ART-mediated viral suppression predicted future atherosclerotic burden among HIV-infected Ugandans	Immune mechanism or inflammation as it relates to hypertension was not addressed

PLWH, people living with HIV; HIV, human immunodeficiency virus; CRP, C-reactive protein; ART, antiretroviral therapy; IL, interleukin; TNFα, tumor necrosis factor alpha; cART, combinational ART; sCD14, soluble CD14; sCD163, soluble CD163; VCAM-1, vascular cell adhesion molecule 1; ICAM-1, intercellular adhesion molecule 1

## Discussion

**Fig. 1:**
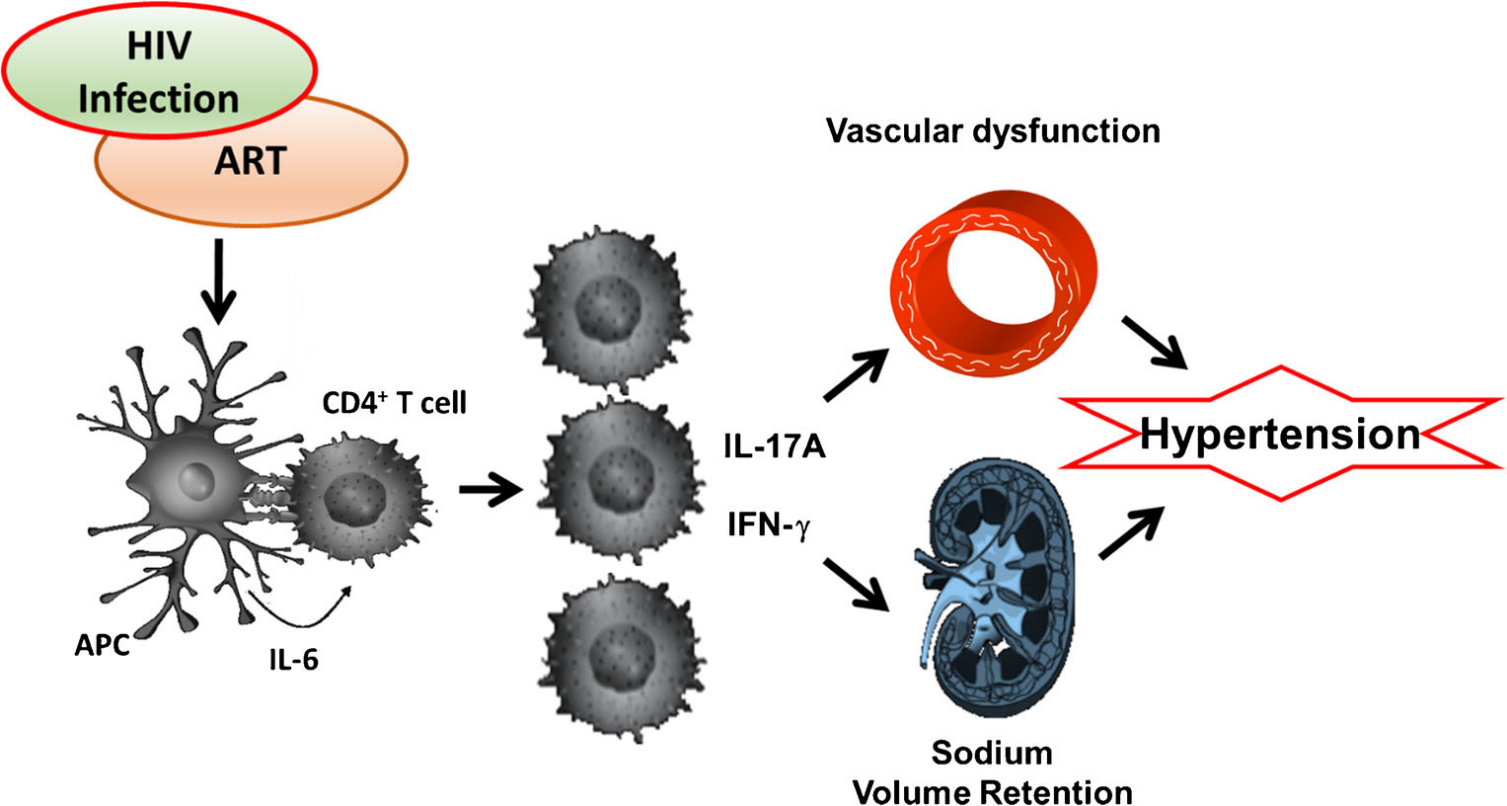
Conceptual schematic of the effect of HIV infection and treatment can activate the immune system leading to hypertension. Viral proteins and/or antiretroviral therapy (ART) activates antigen presenting and T cells which infiltrate the vasculature and the kidneys and release cytokines IL-6, IL-17A, and IFN-γ which promote vascular dysfunction, retention of sodium, and water, leading to hypertension

In the current study, we found that the prevalence of hypertension in PLWH in Sub-Saharan African countries ranged from 2.0 to 50.2% with most cases among those receiving ART. Besides traditional risk factors and the effect of ART on blood pressure, IL-17A, IFN-γ, and CD4^+^ T cells were among the inflammatory parameters associated with hypertension in ART treated PLWH [13••, 56]. The mechanism of interaction between immune activation or inflammation and hypertension in HIV remains elusive and warrants further studies. Our findings and conceptual hypothesis of how immune activation may contribute to hypertension in HIV is shown in Fig. 1. We propose that HIV viral protein and ART interacts with components of the immune system to synergistically induce kidney damage, vascular dysfunction, alterations in sympathetic nervous outflow and lead to hypertension. This process is exacerbated by presence of traditional risk factors exacerbates including obesity, excess dietary salt intake, smoking, and genetic predisposition.

### Prevalence of Hypertension in HIV

The prevalence of hypertension in PLWH varies by population and subgroups even within the same countries. Similar to our findings, Martin-Iguacel et al. and Nguyen et al. reported prevalence ranging from 4 to 54% [57••] and 8.7 to 45.9% among PLWH in low- and middle-income countries [58]. However, in our study, we further segregated prevalence by HIV infection and ART treatment and report magnitude of differences between the groups (Table 1). Higher hypertension prevalence was reported in ART treated PLWH compared with ART-naive participants except in the study by Ogunmola et al. who reported the opposite [22]. This contradictory finding is likely due to differential effects of specific ART regimens and other traditional risk factors differing between study populations. The effect of specific regimens on blood pressure has not yet been well established except for the low to moderate increase attributed to non-nucleoside reverse transcriptase inhibitors (NNRTI’s) and protease inhibitors (PIs) [59, 60]. Prior studies have shown that patients become hypertensive in most cases at least after 2 years of ART and systolic pressure increases further after 5 years of ART [60], and that there is no association between HIV status, ART, and hypertension following short-term follow-up of less than 2 years [22, 61].

### Markers of Immune Activation or Inflammation Associated with Hypertension in HIV

Traditional risk factors associated with hypertension in HIV such as older age, male gender, African-American, higher BMI, central obesity, previous CV events, chronic kidney disease, family history of hypertension and CVD, diabetes, and dyslipidemia have been well studied [57••, 58]. It is also well established that HIV infection and exposure to ART (more than 2 years), through metabolic disturbances and endothelial dysfunction, might have an additional role in the development of hypertension in HIV patients [57••, 58]. However, little is known about the contribution of the innate and adaptive immune system in the development or propagation of hypertension in HIV.

In our systematic review, we found that an improved immune status as determined by higher CD4 T cells was associated with higher hypertension prevalence [17]. However, one study be Okello et al. found that CD4 T cell count of less than 100 was associated with incident hypertension [61]. Martin-Iguacel et al. and Nguyen et al. also reported similar findings [57••, 58]. We found that T cell-derived cytokines IL-17A and IFN-γ were associated with hypertension in ART treated PLWH as reported by Chepchirchir et al. [13••]. These cytokines have been reported to contribute to the genesis of hypertension in the HIV-negative general population and experimental animal studies [6]. Further studies are needed to ascertain the contribution of CD4 T cells and their cytokines IL-17 and IFN-γ in concert with existing traditional risk factors on blood pressure elevation in HIV.

### Immune Activation, Inflammation, and Hypertension

The role of innate and adaptive immunity including specific cell types and cytokines in the development and maintenance of hypertension has been extensively studied in humans and animal models elsewhere [6, 7, 62•, 63–65]. Using multiple experimental animal models, studies have shown that hypertensive stimuli including angiotensin II, aldosterone, endothelin-1, and salt induce activation of immune cells, which infiltrate the vasculature and the kidneys, and release cytokines that induce increased salt and water retention leading to hypertension [6]. This process is mediated in part by increases oxidative stress leading to oxidation of fatty acids and formation of isolevuglandins (IsoLGs) in antigen-presenting cells. IsoLGs activate these cells to produce pro-inflammatory cytokines IL-6, IL-1β, and IL-23, express costimulatory proteins CD80 and CD86, and activate T cells to produce pro-hypertensive cytokines IFN-γ and IL-17A [7]. In humans, plasma isoprostanes, which are produced in concert with IsoLGs, are elevated in hypertension and markedly elevated in patients with resistant hypertension, and IsoLGs are markedly elevated in circulating monocytes of hypertensive patients [7]. IL-17A and IFN-γ have been implicated in the genesis and maintenance of hypertension due in part to their direct effect in causing endothelial dysfunction and renal damage [6, 66]. Thus, similar immuno-pathophysiological mechanisms underlying hypertension in the general population may also contribute to elevated blood pressure in PLWH.

### Potential Mechanisms Leading to Inflammation in HIV

The pathophysiology of HIV-related hypertension seems to emerge from three factors: HIV-related inflammation, HIV-related proteins, and genetic predisposition [59]. It is believed that the HIV viral proteins (negative factor (Nef), transcription proteins (Tat) and glycoprotein 120 (gp-120)) induce hypertension through vascular oxidative stress, smooth myocyte proliferation and migration, and endothelial dysfunction especially in patients with high HIV viral load [59]. Tat, a transactivator protein for HIV replication, is known to be secreted extracellularly by infected cells and has been shown to activate endothelial cells by increasing expression of endothelial-leukocyte adhesion molecules such as intercellular adhesion molecule-1 (ICAM-1), vascular cell adhesion molecule-1 (VCAM-1), and E-selectin which induce initial binding of leukocytes to the blood vessel wall [67]. The levels of soluble ICAM-1 concentration correlate with HIV disease as well as reduction in CD4 count. Tat also induces IL-6 production which increases endothelial permeability [67]. Tat can also suppress the bone morphogenic protein receptor 2 (BMPR-2) responsible for regulating endothelial cell proliferation and survival. This results in increased vascular smooth muscle proliferation and activation of endothelial cells leading to pulmonary arterial hypertension (PAH) [68]. In HIV-positive patients, Tat has been shown to increase the transcription of IL-17 and secretion by T cells causing a pro-inflammatory milieu and has been associated with a devastating immune reconstitution inflammatory syndrome (IRIS) in the brain [69]. The HIV Gp120 is found on the surface of the HIV envelope and can also be found in circulation from viral turn over [70]. Gp120 stimulates monocytes and macrophages to release pro-inflammatory cytokines, promotes an increase in markers of apoptosis, and stimulates the secretion of endothelin-1, a potent vasoconstrictor [68]. It induces endothelial apoptosis by interacting with CXCR4 also expressed on endothelial cells [70]. Gp120 and TNF-α synergistically decrease eNOS and NO in human coronary artery endothelial cells [70].

DCs, the most potent antigen-presenting cells of the innate immune system, have been shown to interact with components of HIV-1 both intrinsically (following fusion of cellular and viral membrane) and extrinsically (prior to being infected) through pathogen recognition receptors (PRRs) resulting in activation of the adaptive immune system [71]. Through their CD28/B7 ligands CD80 and CD86 (also expressed on monocytes and B cells), DCs provide a costimulatory signal necessary for T cell activation and survival. In hypertension, there is an increase in B7 ligand expression leading to T cell activation [5, 72]. An important element in the activation of T cells by DCs and other cells in hypertension is antigen presentation. It is unclear what antigens are presented to T cells in HIV and it is not known if IsoLG-adducted peptides might play a role in hypertension associated with HIV.

Monocyte activation has an important role in HIV-infected persons on ART. Monocytes are chronically activated during HIV infection and it is now evident that inflammatory mediators produced by monocytes (especially IL-6), independent of T cell activation, also predict serious non-AIDS events (SNAEs) in virologically suppressed HIV-infected persons treated with ART [73]. IL-6 production was higher in monocytes than other cells and was associated with increased odds of SNAE and mortality but not the percentage of activated CD4 and CD8 T cells (those expressing CD38 or CD38 and HLA-DR) [73]. Infected monocytes, especially intermediate monocytes expressing CD14^+^ and CD16^+^, produce more pro-inflammatory cytokines as compared to their counterpart. They adhere to endothelial cells and transmigrate into the sub endothelial area where their pro-inflammatory activity is increased. It is evident that in HIV, monocytes and macrophages have a reduced phagocytic activity and they age prematurely [70]. Monocytes/macrophages have been implicated in the pathogenesis of hypertension in the general population and experimental animal models [5].

Several mechanisms of immune cell activation have been reported in HIV. First, there is direct binding of the HIV envelope protein gp120/160 to CD4 and/or C-C chemokine receptor type 5 (CCR5) which down-modulates the expression of CD3^+^ T cell receptor in infected cells resulting in cell death; second, there is an interaction between HIV viral particles and plasmacytoid dendritic cells (DCs) through Toll like receptor (TLR) stimulation which activates CD8^+^ cytotoxic cells; third, there is microbial translocation from a leaky gut into the circulation leading to a generalized systemic inflammatory process [74]. Also important is the dysfunction of T regulatory cells and immune-senescence resulting in an uncontrolled over activation of immune cells in HIV [74]. The resultant low-grade chronic inflammation which persists even after initiation of ART is associated with hypertension and cardiovascular disease [75, 76].

IL-6 production by monocytes and macrophages is increased following HIV infection and is a valuable prognostic marker for disease progression [77, 78]. Soluble CD14 (sCD14) produced from monocytes and macrophages is also elevated in HIV-infected persons regardless of treatment status and is an independent predictor of mortality [73]. Anzinger et al. reported that increased microbial translocation from the gut resulting from depletion of CD4^+^ T cells in acute HIV infection results in monocyte activation and higher sCD14 [73]. The expression of programmed death-1 (PD-1), a member of the B7:28 inhibitory molecules, is rapidly increased on CD4^+^ and CD8^+^ T cells, B cells, natural killer cells, and monocytes as they interact with PD-L1, its ligand on antigen-presenting cells such that engagement on virus-infected cells leads to impaired generation of effector T cell responses [74]. Other markers which have been associated with immune activation in HIV include increased expression of CD28^−^, CD57^+^ and reduced expression of CD127 on CD4^+^ and CD8^+^ T cells [79, 80].

In our analysis, we found HIV infection alone is associated with an inflammatory milieu involving higher levels of IL-6, CRP, VCAM-1 ICAM-1, and intermediate monocytes (CD14^+^16^+^) among studies conducted in Sub-Saharan African cohorts [17, 55]. There is a generalized systemic immune activation following HIV infection which subsides but still persists at low-grade or subclinical following initiation of ART involving activation of both CD4^+^ and CD8^+^ T cells expressing high levels of CD38 [74]. Other T cell activation markers such as human leukocyte antigen–antigen D related (HLA-DR) as well as increases in pro-inflammatory cytokines tumor necrosis factor alpha (TNF-α), interleukin 6 (IL-6), and interleukin 1 beta (IL-1β) have been reported in PLWH in the western countries [74]. Increased expression of CD38 alone or in concert with HLA-DR^+^ in T cells is a marker of disease progression and mortality in some cases correlating directly with HIV viral load and indirectly with CD4^+^ count [74]. This continuous immune activation and inflammation has been proposed to contribute to endothelial dysfunction, hypertension, and cardiovascular disease [81].

### Limitations, Conclusion, and Future Perspectives

Almost all studies except one [13••] were not designed to compare blood pressure or hypertension and inflammation in PLWH as the primary outcomes. There is need for additional prospective and mechanistic studies to establish the relationship between inflammation and hypertension in HIV infection, and ART. Most studies addressing the mechanistic role of inflammation in the genesis and progression of hypertension have been conducted in murine models and humans in the HIV-negative context. The inflammatory milieu in HIV infection is obviously complex and very different from the general population and further studies are needed to determine the specific contribution of the various attributes in HIV including the viral proteins and ART on inducing inflammation associated with hypertension.
